# Glycative stress inhibits hypertrophy and impairs cell membrane integrity in overloaded mouse skeletal muscle

**DOI:** 10.1002/jcsm.13444

**Published:** 2024-04-04

**Authors:** Tatsuro Egawa, Takeshi Ogawa, Takumi Yokokawa, Kohei Kido, Ryota Iyama, Haiyu Zhao, Eriko Kurogi, Katsumasa Goto, Tatsuya Hayashi

**Affiliations:** ^1^ Laboratory of Health and Exercise Sciences, Graduate School of Human and Environmental Studies Kyoto University Kyoto Japan; ^2^ Laboratory of Sports and Exercise Medicine, Graduate School of Human and Environmental Studies Kyoto University Kyoto Japan; ^3^ Division of Food Science and Biotechnology, Graduate School of Agriculture Kyoto University Kyoto Japan; ^4^ Health and Medical Research Institute National Institute of Advanced Industrial Science and Technology (AIST) Kagawa Japan; ^5^ Institute for Physical Activity Fukuoka University Fukuoka Japan; ^6^ Laboratory of Physiology, Graduate School of Health Sciences Toyohashi SOZO University Toyohashi Japan

**Keywords:** Advanced glycation end products, Muscle dysfunction, Muscle hypertrophy, Protein synthesis, Sarcolemma integrity

## Abstract

**Background:**

Glycative stress, characterized by the formation and accumulation of advanced glycation end products (AGEs) associated with protein glycation reactions, has been implicated in inducing a decline of muscle function. Although the inverse correlation between glycative stress and muscle mass and strength has been demonstrated, the underlying molecular mechanisms are not fully understood. This study aimed to elucidate how glycative stress affects the skeletal muscle, particularly the adaptive muscle response to hypertrophic stimuli and its molecular mechanism.

**Methods:**

Male C57BL/6NCr mice were randomly divided into the following two groups: the bovine serum albumin (BSA)‐treated and AGE‐treated groups. Mice in the AGE‐treated group were intraperitoneally administered AGEs (0.5 mg/g) once daily, whereas those in the BSA‐treated group received an equal amount of BSA (0.5 mg/g) as the vehicle control. After 7 days of continuous administration, the right leg plantaris muscle of mice in each group underwent functional overload treatment by synergist ablation for 7 days to induce muscle hypertrophy. In in vitro studies, cultured C2C12 myocytes were treated with AGEs (1 mg/mL) to examine cell adhesion and cell membrane permeability.

**Results:**

Continuous AGE administration increased the levels of fluorescent AGEs, Nε‐(carboxymethyl) lysine, and methylglyoxal‐derived hydroimidazolone‐1 in both plasma and skeletal muscle. Plantaris muscle weight, muscle fibre cross‐sectional area, protein synthesis rate, and the number of myonuclei increased with functional overload in both groups; however, the increase was significantly reduced by AGE treatment. Some muscles of AGE‐treated mice were destroyed by functional overload. Proteomic analysis was performed to explore the mechanisms of muscle hypertrophy suppression and myofibre destruction by AGEs. When principal component analysis was performed on 4659 data obtained by proteomic analysis, AGE treatment was observed to affect protein expression only in functionally overloaded muscles. Enrichment analysis of the 436 proteins extracted using the K‐means method further identified a group of proteins involved in cell adhesion. Consistent with this finding, dystrophin–glycoprotein complex proteins and cell adhesion‐related proteins were confirmed to increase with functional overload; however, this was attenuated by AGE treatment. Additionally, the treatment of C2C12 muscle cells with AGEs inhibited their ability to adhere and increased cell membrane permeability.

**Conclusions:**

This study indicates that glycative stress may be a novel pathogenic factor in skeletal muscle dysfunctions by causing loss of membrane integrity and preventing muscle mass gain.

## Introduction

Glycation is a non‐enzymatic post‐translational modification of proteins primarily occurring between amino acids and reducing sugars, and proteins are eventually modified to advanced glycation end products (AGEs). AGE‐modified proteins lose their normal function, as well as activate inflammatory signalling and generate oxidative stress by binding to the receptor for AGEs (RAGE), which adversely affects physical functions. ‘Glycative stress’ refers to the overall concept of the deleterious effects on the body caused by glycation and its associated reactions.^1^ AGE‐modified proteins increase with aging and are involved in the development of age‐related diseases, including cancer, Alzheimer's disease, vascular diseases, osteoporosis, complications of diabetes, and sarcopenia.^2^, ^3^ Therefore, glycative stress, along with oxidative stress, has been recognized as an aging‐promoting factor.

Glycative stress has been implicated in the aging of skeletal muscle, the largest metabolic and endocrine organ in the body. Several epidemiologic studies have shown that AGE accumulation is associated with reduced muscle mass and strength and walking disability,^4^ strongly suggesting that glycative stress is a trigger for sarcopenia. Mechanistically, glycation of muscle contractile proteins, including myosin, actin, and tropomyosin and extracellular matrix, as well as AGE‐induced motor neurotransmission impairment are critical factors for the deterioration of muscle contractile properties.^5^ Our previous studies have shown that long‐term exposure to glycative stress suppresses skeletal muscle development accompanied by the dysfunction of myogenic capacity and insulin‐like growth factor signalling^6^, ^7^ and upregulates inflammatory cytokine expression in mice.^8^ Furthermore, the inhibition of RAGE prevented disuse‐ and cancer cachexia‐induced muscle wasting in mice.^9^, ^10^ Moreover, other investigators have shown that glycative stress induces skeletal muscle atrophy in diabetic mice^11^ and that glyceraldehyde‐derived AGEs, known as toxic AGEs, induce muscle death in myoblast cells.^12^ Interestingly, recent studies have shown an association between AGE accumulation and impaired motor and skeletal muscle function in children and adolescents.^13^, ^14^ Therefore, glycative stress can affect skeletal muscle function regardless of age.

These findings suggest that glycative stress has a negative impact on normal skeletal muscle function maintenance in both aged and young muscles. However, the effect of glycative stress on the muscle hypertrophic response remains unclear. Based on the abovementioned evidence, we hypothesized that glycative stress would impair muscle hypertrophy. Therefore, this study aimed to clarify how glycative stress affects muscle adaptations to hypertrophic stimuli. For this purpose, synergist ablation‐induced functional muscle overload was applied to mice subjected to glycative stress by AGE injection. We noted that muscle hypertrophy was suppressed in AGE‐treated mice compared with that in control mice. Moreover, muscle destruction was unexpectedly observed in some mice. To investigate the mechanism, proteomic analysis was performed and showed that AGEs caused dysfunctions in muscle cell membrane integrity.

## Methods

### Preparation of advanced glycation end products

AGEs were prepared as previously described.^15^ In brief, 50 mg/mL bovine serum albumin (BSA) (Nacalai Tesque, Kyoto, Japan) was incubated with 0.1 M glyceraldehyde (Sigma‐Aldrich Co., St. Louis, MO, USA) in 0.2 M phosphate buffer (pH, 7.4) under sterile conditions at 37°C for 7 days. The unincorporated glyceraldehyde was subsequently removed by dialysis. Non‐glycated BSA was incubated under the same conditions except in the absence of glyceraldehyde as a negative control.

### Animals and treatment

All animal protocols were performed in accordance with the Guide for the Care and Use of Laboratory Animals by the National Institutes of Health (Bethesda, MD, USA) and were approved by the Kyoto University Graduate School of Human and Environmental Studies (approval number: 29‐A‐2).

**Figure 1:**
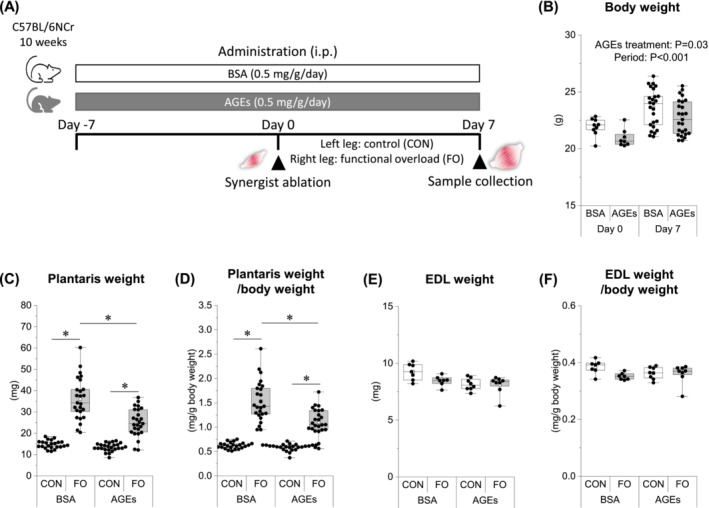
AGE administration prevents overload‐induced muscle mass gain. (A) Experimental design for evaluating the effect of AGEs on overload‐induced muscle hypertrophy. (B) Body weight before (day 0) and after (day 7) the overload surgery. (C) Plantaris weight on day 7. (D) Plamtaris weight‐normalized body weight on day 7. (E) Extensor digitorum longus muscle (EDL) weight on day 7. (F) EDL weight‐normalized body weight on day 7. Data are shown as box plots. White square indicates mean values. *N* = 7–25 mice/group. Individual data points are indicated on the graph. Statistical significance is analysed using two‐way ANOVA with AGE treatment and period as main factors. *P < 0.05 with simple effects tests. AGEs, advanced glycation end products; ANOVA, analysis of variance; BSA, bovine serum albumin; CON, control muscle; FO, functional overloaded muscle.

Male C57Bl/6NCr mice (9 weeks old) were purchased from Shimizu Breeding Laboratories (Kyoto, Japan). Mice were housed in a room maintained at 22–24°C with a 12‐h light–12‐h dark cycle and fed a standard chow. The experimental schedule is shown in Figure 1A. After 1‐week acclimatization, mice were randomly divided into the following two groups: the BSA‐treated (BSA; *n* = 40) and AGE‐treated groups (AGEs; *n* = 42). Mice in the AGE‐treated group received AGEs (0.5 mg/g) intraperitoneally, whereas those in the BSA‐treated group received the same amount of non‐glycated BSA (0.5 mg/g) as a vehicle control. The administration was continuous once daily until sample collection. On the day after 7 days of continuous administration (day 0), some mice (BSA, *n* = 8; AGEs, n = 8) were killed for blood sampling, and the others (BSA, *n* = 32; AGEs, *n* = 34) underwent overload surgery. The day after 7 days of overload surgery (day 7), body weights were measured, and plantaris and extensor digitorum longus (EDL) muscles and blood samples were collected. Plantaris and EDL muscles were measured for wet weight and stored at −80°C until the experiment.

### Overload surgery

To induce overload‐induced hypertrophy, the plantaris muscle of the right hindlimb was functionally overloaded (FO) following synergist ablation of the soleus and gastrocnemius muscles. Briefly, the soleus and gastrocnemius muscles were cut and removed under anaesthesia with an intraperitoneal injection of mixtures of medetomidine hydrochloride (0.75 mg/kg), midazolam (4 mg/kg), and butorphanol tartrate (5 mg/kg); subsequently, the skin was sutured, and mice were placed in the cages. The contralateral left hindlimb was left intact as an internal control (CON).

### Motor performance test

The grip strength test and rotarod test were conducted on the day prior to the end of the experiment. Grip strength was measured using a force transducer (DS2‐50N; IMADA, Toyohashi, Japan) following the published protocol.^6^ Briefly, mice were placed on the horizontal mesh with all four limbs and were then gently pulled back until their grip was released. Three consecutive measurements were taken within 1 min and were averaged to determine the mean grip strength. The data were normalized to body weight and expressed as N/g.

The rotarod test was conducted following the previously described protocol.^16^ The mouse was allowed to adapt for 30 s at 5 rpm, after which the rotation speed was increased by 5 rpm every 30 s until the mouse fell, and the rotation speed at that point was recorded.

### Measurement of advanced glycation end products

The AGE content in administered substances, blood, and muscle samples was measured using fluorescence intensity or the enzyme‐linked immunosorbent assay (ELISA). AGE fluorescence intensity was determined at a 360 nm excitation wavelength and a 460 nm emission wavelength using Synergy LX Multi‐Mode Microplate Reader (BioTek, Winooski, VT, USA). The fluorescence intensity levels were normalized to protein concentration and expressed in arbitrary units. The OxiSelect Nε‐(carboxymethyl) lysine (CML) Competitive ELISA Kit (STA‐816, Cell Biolabs, San Diego, CA, USA), OxiSelectTM Nε‐(carboxyethyl) lysine (CEL) Competitive ELISA Kit (STA‐813, Cell Biolabs), and OxiSelect Methylglyoxal (MG) Competitive ELISA Kit (STA‐811, Cell Biolabs) were used to determine the quantitative AGEs content. The CML, CEL, and MG content in muscles were normalized to protein concentration and expressed as μg/mg protein. The protein concentration was measured in duplicate using the Bradford technique.

### Immunohistochemistry

For immunohistochemistry, the plantaris muscles were embedded in FSC 22 Frozen Section Media (Leica Biosystems, Wetzlar, Germany) and frozen in liquid nitrogen‐cooled isopentane and stored at −80°C. Seven‐micrometre‐thick sections from the plantaris muscles of the midbelly portion were obtained using a cryostat (Leica Biosystems) at −25°C. For cell membrane staining, sections were boiled in phosphate‐buffered saline (PBS) solution, blocked with Blocking One Histo (Nacalai Tesque, Kyoto, Japan) for 30 min, and incubated with rabbit anti‐laminin IgG antibody (L9393, Sigma‐Aldrich, St. Louis, MO, USA, diluted with 1:200) overnight at 4°C. The sections were subsequently incubated with Alexa Fluor 488‐conjugated anti‐rabbit IgG (111‐545‐003, Jackson Laboratory, Bar Harbour, ME, USA, diluted with 1:200) for 1 h at room temperature. Subsequently, nuclei were stained with 4′,6‐diamidino‐2‐phenylindole dihydrochloride (DAPI) (Dojindo Laboratories, Kumamoto, Japan) for 15 min and mounted with VECTASHIELD Mounting Medium (Vector Laboratories, Burlingame, CA, USA).

Sections were imaged at 10× magnification using a microscope (CKX53, OLYMPUS, Tokyo, Japan). To determine the muscle fibre cross‐sectional area (CSA) and myonucleus count, digitized images were analysed using ImageJ software. The same images used to determine the CSA were used to determine the number of myonuclei per fibre. DAPI‐stained nuclei whose centre was within the laminin boundary were counted as myonuclei. To determine the CSA and myonucleus count, at least 100 fibres per image were measured.

### Proteomics

Proteomics was performed by Kazusa DNA Research Institute (Chiba, Japan). Briefly, the muscle homogenate was precipitated in acetonitrile (ACN), and the precipitate was extracted in 0.5% sodium dodecanoate and 100 mM Tris–HCl (pH, 8.5). The extracted proteins were adjusted to 1 mg/mL with 0.5% sodium dodecanoate and 100 mM Tris–HCl (pH, 8.5). The 20‐μg protein extract was treated with 10 mM dithiothreitol at 50°C for 30 min and subsequently subjected to alkylation with 30 mM iodoacetamide in the dark at room temperature for 30 min. The iodoacetamide reaction was stopped with 60 mM cysteine for 10 min. The mixture was diluted with 150 μL of 50 mM ammonium bicarbonate and digested by adding 0.4 μg trypsin and 0.4 μg Lys‐C overnight at 37°C. The digested sample was acidified with 30 μL of 5% trifluoroacetic acid, vortexed for 5 min, and centrifuged at 15 000× *g* for 10 min. The supernatant was desalted using C18‐StageTips and subsequently dried in a centrifugal evaporator. The dried peptides were redissolved in 3% ACN and 0.1% formic acid. The redissolved peptides were measured using the BCA protein assay technique and adjusted to 0.1 mg/mL with 3% ACN and 0.1% formic acid.

Peptides were directly injected onto a 75 μm × 25 cm PicoFrit emitter (New Objective, Woburn, MA, USA) packed in house with C18 core‐shell particles (CAPCELL CORE MP 2.7 μm, 160 Å material; Osaka Soda Co., Ltd., Osaka, Japan) and subsequently separated using an UltiMate 3000 RSLC nanoLC system (Thermo Fisher Scientific, Waltham, MA, USA). Peptides eluting from the column were analysed on a Q Exactive HF‐X (Thermo Fisher Scientific). MS1 spectra were collected in the range of 495–785 *m*/*z* at 30 000 resolution to set an automatic gain control (AGC) target of 3 × 10^6^. MS2 spectra were collected in the range of >200 *m/z* at 15 000 resolution with stepped normalized collision energies of 24, 26, and 28 to set an AGC target of 3 × 10^6^.

Data were further analysed using Scaffold‐DIA software (Proteome Software, Portland, OR, USA) with Prosit‐Derived Spectral Libraries for Scaffold DIA Searches (https://support.proteomesoftware.com/hc/en‐us/articles/360035151172‐Prosit‐Derived‐Spectral‐Libraries‐for‐Scaffold‐DIA‐Searches).

### Data analysis of proteomics

For proteomic datasets, the intensities of the respective protein groups were analysed using Perseus version 1.6.10.45 (https://maxquant.net/perseus/) and R version 3.6.2 (https://cran.r‐project.org/bin/windows/base/old/3.6.2/). Raw intensities were log10 logarithmized and normalized by *Z*‐score. Pearson correlation analysis and principal component analysis (PCA) were performed following data preprocessing. Subsequently, two‐way analysis of variance (ANOVA) was performed with overload surgery (CON or FO) and AGE treatment (BSA or AGEs) as main factors. Protein groups with significant interactions following two‐way ANOVA were extracted and analysed by K‐means clustering. Gene ontology (GO) enrichment analysis was performed on potentially interesting clusters using the DAVID (https://david.ncifcrf.gov) online tool. Kyoto Encyclopedia of Genes and Genomes (KEGG) pathway names and the Gene Ontology Biological Process (GOBP), Cellular Component (GOCC), and Molecular Function (GOMF) names were annotated to the corresponding protein groups on the basis of UniProt accession numbers.

### Western blotting

Muscle samples for western blotting were prepared as previously described.^6^ Briefly, the muscles were homogenized in ice‐cold lysis buffer (1:40 wt/vol) containing 20 mM Tris–HCl (pH, 7.4), 1% Triton X, 50 mM NaCl, 250 mM sucrose, 50 mM NaF, 5 mM sodium pyrophosphate, 2 mM dithiothreitol, 4 mg/L leupeptin, 50 mg/L trypsin inhibitor, 0.1 mM benzamidine, 1 mmol/L Na_3_VO_4_, and 0.5 mM phenylmethylsulfonyl fluoride; the homogenates were subsequently centrifuged at 16 000× *g* for 30 min at 4°C, and the supernatants were collected.

Sample proteins (10 μg) were separated on polyacrylamide gels and transferred to polyvinylidene difluoride membranes. Membranes were blocked using EveryBlot blocking buffer (Bio‐Rad Laboratories, Hercules, CA, USA) for 5 min and subsequently incubated overnight at 4°C using the following commercially available antibodies: α‐dystroglycan (IIH6 C4‐s, Developmental Studies Hybridoma Bank [DSHB], Iowa City, IA, USA), α‐sarcoglycan (IVD3^1^ A9, DSHB), carcinoembryonic‐antigen–related cell‐adhesion molecule 1 (CEACAM1) (sc‐365126, Santa Cruz, CA, USA), junctional adhesion molecule C (JAM‐C) (AF1213, R&D Systems, Minneapolis, MN, USA), VE‐cadherin (sc‐9989, Santa Cruz), integrin β3 (1B5, DSHB), dystrophin (8B11, DSHB), and tetraspanin‐4 (NBP2‐93365, Novus Biologicals, Santa Cruz, CA, USA), mTOR (2972, Cell Signaling Technology, Danvers, MA, USA), phospho‐mTOR Ser^2448^ (2971, Cell Signaling Technology), p70 S6 kinase (9202, Cell Signaling Technology), phospho‐p70 S6 kinase Thr^389^ (9234, Cell Signaling Technology), 4E‐BP1 (9452, Cell Signaling Technology), and phospho‐4E‐BP1 Thr^37/46^ (9459, Cell Signaling Technology). Membranes were then incubated with anti‐mouse IgG (7074, Cell Signaling Technology, Danvers, MA, USA) or anti‐rabbit IgG (7076, Cell Signaling Technology) coupled to horseradish peroxidase for 1 h at room temperature. Each primary and secondary antibody was diluted at 1:5000 and 1:10 000, respectively. Protein bands were visualized using Chemi‐Lumi One Ultra (Nacalai Tesque) and a bioimaging analyser (LuminoGraph II, ATTO, Tokyo, Japan). Equal protein loading and transfer efficiency were verified by Coomassie Brilliant Blue staining of the membranes.

### Muscle protein synthesis measurement

Muscle protein synthesis was measured using the in vivo surface sensing of translation (SUnSET) method, as previously described.^17^, ^18^ Briefly, mice were intraperitoneally injected with puromycin (0.04 μmol/g body weight) (Nacalai Tesque). Thirty minutes following injection, the plantaris muscles were immediately removed and frozen in liquid nitrogen. Following homogenization and centrifugation at 16 000× *g* for 30 min at 4°C, supernatants were collected and processed for western blot analysis. A mouse monoclonal anti‐puromycin antibody (MABE343, Millipore, Cambridge, MA, USA) was used for detecting puromycin incorporation, which was quantified as the sum of all protein band intensities in the western blot analysis.

### Cell adhesion assay

Mouse myoblast C2C12 cells (DS Pharma Biomedical, Osaka, Japan) were incubated with Dulbecco's modified Eagle's medium (DMEM, Invitrogen, Rockville, MD, USA) containing BSA or AGEs at 1 or 5 mg/mL concentration for 60 min at 37°C in a centrifuge tube. Cell adhesion capacity was subsequently measured using a CytoSelect 48‐well Cell Adhesion Assay (Cell Biolabs, San Diego, CA, USA) according to the manufacturer's protocol. Briefly, the C2C12 myoblast suspension was seeded onto a type I collagen‐coated 48‐well culture plate at a density of 2.0 × 10^4^ per well. Sixty minutes after seeding, the media was aspirated, and each well was washed four times with Dulbecco's modified PBS. The myoblasts were subsequently stained with 0.2% crystal violet solution for 10 min. After washing with water, three fields were randomly selected from each well, and the stained myoblasts were counted under a microscope.

### Cytotoxicity detection assay

Cell culture was performed as previously described.^19^ C2C12 myoblasts were cultured in a 48‐well culture plate at a density of 2.0 × 10^4^ per well. Cells were maintained in growth medium consisting of DMEM supplemented with 10% heat‐inactivated fetal bovine serum for proliferation. After reaching confluence, the culture medium was changed to DMEM containing BSA or AGEs at 1 or 5 mg/mL concentration, and cells were incubated for 60 min. Then, the amount of lactate dehydrogenase (LDH) in the supernatant of the cell culture medium was measured using an LDH Cytotoxicity Detection Kit (Takara Bio, Kusatsu, Japan) according to the manufacturer's protocol. The cytotoxicity value was expressed as the percentage of cells stimulated with Triton X‐100.

### Cell injury and cell membrane permeability assay

Cell injury methods were performed as previously described,^20^ with some modifications. Briefly, C2C12 myoblasts were cultured in a 24‐well culture plate at a density of 2.0 × 10^4^ per well. Cells were maintained in growth medium consisting of DMEM supplemented with 10% heat‐inactivated fetal bovine serum for proliferation. After reaching confluence, the culture medium was changed to the same amount of differentiation medium consisting of DMEM supplemented with 2% heat‐inactivated horse serum to initiate the differentiation. Five days after the initiation of differentiation, myotubes were incubated with differentiation medium containing BSA or AGEs at 1 mg/mL concentration. Forty‐eight hours following incubation, myotubes were injured by rolling 5 mg of glass beads (G8772, Sigma‐Aldrich, St. Louis, MO, USA) over the cells at room temperature by manually tilting the plates back and forth 10 times at a 30° angle in the presence of 1 mg/mL fluorescein isothiocyanate (FITC)‐lysine‐dextran (4 kDa) (Cosmo Bio, Tokyo, Japan). After removing the glass beads and FITC‐dextran by washing with PBS, the myotubes were fixed with 4% paraformaldehyde, and three fields were randomly selected from each well and imaged under a microscope. The fluorescence intensity of FITC‐positive myotubes was measured using ImageJ software.

### Statistical analysis

For data not related to the proteomic datasets, quantitative data were presented as box plots with medians as horizontal lines, 25th and 75th percentiles as boxes, and the minimum or maximum values as whiskers. Statistical significance was analysed using two‐way ANOVA with AGE treatment (BSA or AGEs) and period (day 0 or day 7) or overload surgery (CON or OL) as main factors. Post hoc multiple comparison tests between factors were performed following two‐way ANOVA. Post hoc analysis was conducted using Tukey–Kramer's test. The Mann–Whitney *U* test was used to compare differences between two independent groups. Differences between groups were considered statistically significant at *P* < 0.05. Statistical analysis was performed using BellCurve for Excel software (Social Survey Research Information, Tokyo, Japan).

## Results

### Plasma and muscle advanced glycation end products levels increased with advanced glycation end products administration

Tables S1 and S2 show the concentrations of AGEs in the administered substances, plasma, and plantaris muscles, respectively. The fluorescent AGEs level of the prepared glyceraldehyde‐derived AGEs‐BSA was 36 times higher than that of the BSA control. Additionally, CML, CEL, and methylglyoxal‐derived hydroimidazolone‐1 (MG‐H1) were detected (Table S1). Continuous administration of AGEs resulted in a significant increase in plasma fluorescent AGEs, CML, CEL, and methylglyoxal‐derived hydroimidazolone 1 (MG‐H1) levels, up to approximately four times that of the BSA‐treated group. This increase was sustained even during overload (Table S2). Additionally, accumulation of fluorescent AGEs, CML, and MG‐H1 was observed in the plantaris muscles (Table S2).

### Advanced glycation end products prevented overload‐induced muscle mass gain and impaired motor function

Body weight increased over time in both groups but was lower in the AGE‐treated group than that in the BSA‐treated group (Figure 1B). Both plantaris weight and body weight‐normalized muscle weight increased with functional overload in both groups; however, the increase was significantly reduced by AGE treatment (Figure 1C,D). There was no change in EDL weight with any stumulation (Figure 1E,F).

To evaluate the impact of AGEs on motor function, we performed muscle strength test and rotarod test. There was no difference in four‐limb muscle strength between the AGE‐ and BSA‐treated groups (Figure S1A). However, the rotarod performance of the AGE‐treated group was lower (Figure S1B).

### Advanced glycation end products inhibited overload‐induced increase in skeletal muscle protein synthesis

**Figure 2:**
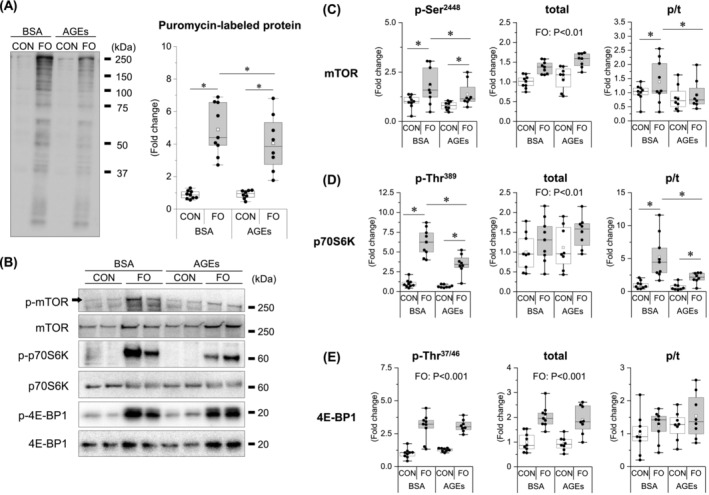
AGE administration inhibits the overload‐induced increase in skeletal muscle protein synthesis signalling. (A) Protein synthesis rate. Protein synthesis rate is measured using the surface sensing of translation (SUnSET) technique. (B) Immunoblots of protein synthesis signalling molecules. (C) Mechanistic target of rapamycin (mTOR). (D) 70‐kDa ribosomal protein s6 kinase (p70S6K). (E) 4E‐binding protein 1 (4E‐BP1). Data are shown as box plots. White square indicates mean values. *N* = 8–9 mice/group. Individual data points are indicated on the graph. Representative immunoblots are shown. Statistical significance is analysed using two‐way ANOVA with AGE treatment and overload surgery as main factors. *P < 0.05 with simple effects tests.

To investigate the impact of AGEs on muscle protein synthesis, we measured puromycin uptake as an index of protein synthetic capacity using the SUnSET method. Furthermore, we assessed the phosphorylation status of mTOR, p70S6K, and 4E‐BP1, which are signalling molecules involved in protein synthesis. The levels of puromycin‐labelled protein were increased by functional overload in both groups. However, AGE treatment significantly reduced the enhancement (Figure 2A). Functional overload enhanced the phosphorylation of mTOR Ser^2448^ and p70S6K Thr^389^, as well as their phosphorylation ratio to total. However, AGEs treatment alleviated this effect (Figure 2C,D). While both the phosphorylation and total expression of 4E‐BP1 increased with functional loading, the phosphorylation ratio remained unchanged (Figure 2E).

### Advanced glycation end products suppressed overload‐induced muscle hypertrophy and induced muscle destruction

**Figure 3:**
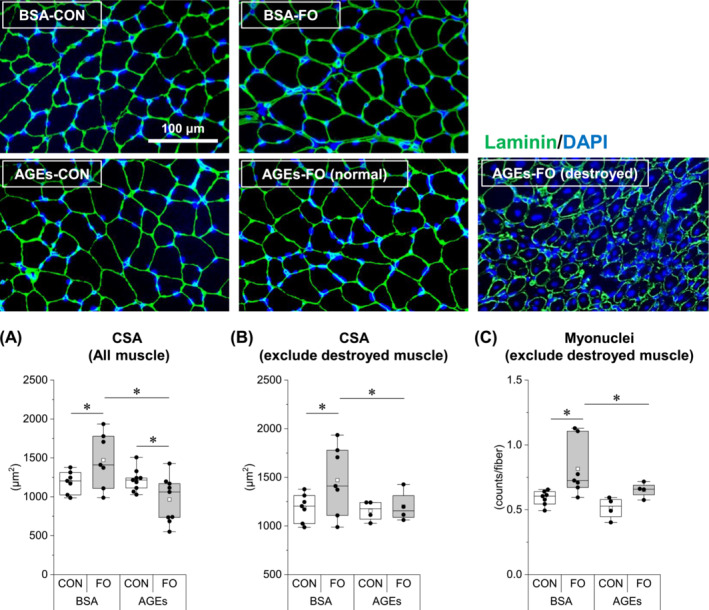
AGE administration suppresses overload‐induced muscle hypertrophy and induces muscle destruction. (A) Muscle fibre cross‐sectional area (CSA) of all muscles. (B) CSA of muscles excluding destroyed muscles. (C) Myonuclei of muscles excluding destroyed muscles. Data are shown as box plots. White square indicates mean values. *N* = 4–9 mice/group. Individual data points are indicated on the graph. Representative immunofluorescence images are shown. Statistical significance is analysed using two‐way ANOVA with AGE treatment and overload surgery as main factors. *P < 0.05 with simple effects tests.

To clarify whether AGEs affect muscle fibre hypertrophy, the muscle fibre CSA and myonuclear number of each group were compared. Consistent with the results of muscle mass and protein synthesis, the CSA was increased by functional overload, and its increase was completely suppressed by AGE treatment (Figure 3A). Surprisingly, however, the muscles of five out of nine AGE‐treated mice were destroyed by functional overload, as shown in the image. Because damaged muscle fibres greatly affect CSA, we next evaluated excluding them. As a result, even when the mice with destroyed muscles were excluded, the average values of the CSA did not change the trend (Figure 3B), suggesting that changes in CSA occur independently of muscle fibre destruction. As the number of myonuclei cannot be properly counted in destroyed muscles, we counted the number of myonuclei per muscle fibre excluding mice with destroyed muscle and observed that AGE treatment suppressed the overload‐induced increase in the number of myonuclei (Figure 3C).

### Cell membrane integrity‐related proteins were affected by advanced glycation end products in overloaded skeletal muscle

**Figure 4:**
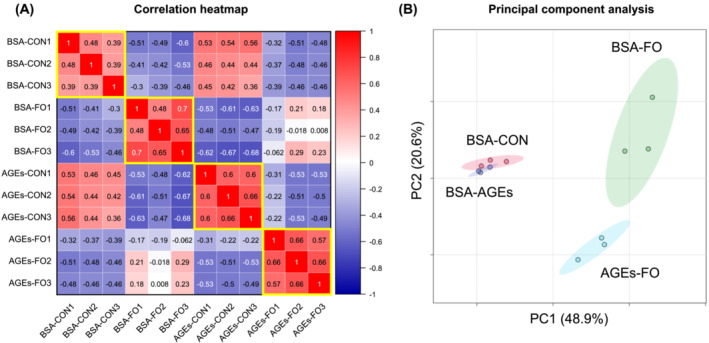
Proteomic data following overload‐induced muscle hypertrophy. (A) Pearson correlation analysis of the identified protein groups of the plantaris muscle of the four groups. Positive and negative correlation are shown in red and blue, respectively, along with the correlation values. Yellow frames indicate correlations within each group. (B) Principal component analysis (PCA) plot of the identified protein groups of the plantaris muscle of four groups. *N* = 3 mice/group. Elliptical areas represent 95% confidence regions.

To explore the mechanisms of muscle hypertrophy inhibition and myofibre destruction by AGEs, proteomic analysis was performed. Proteomic analysis detected 4659 proteins, and then data preprocessing, Pearson correlation analysis, and PCA were performed. Pearson correlation analysis shows the correlation of 4659 protein expressions between each group, and this showed that there was a positive correlation of protein expression levels within each group (Figure 4A, yellow frame), indicating the high biological reproducibility and reliability of the proteomic datasets. PCA showed that principal component 1 (PC1) and principal component 2 (PC2) captured 48.9% and 20.6% of the variation in the dataset, respectively (Figure 4B). The 95% confidence regions of BSA‐FO and AGEs‐FO were clearly distinguished, whereas those of BSA‐CON and AGEs‐CON were overlapped (Figure 4B). This indicates that AGE treatment affected the protein expressions only in the functional‐overloaded muscle.

**Figure 5:**
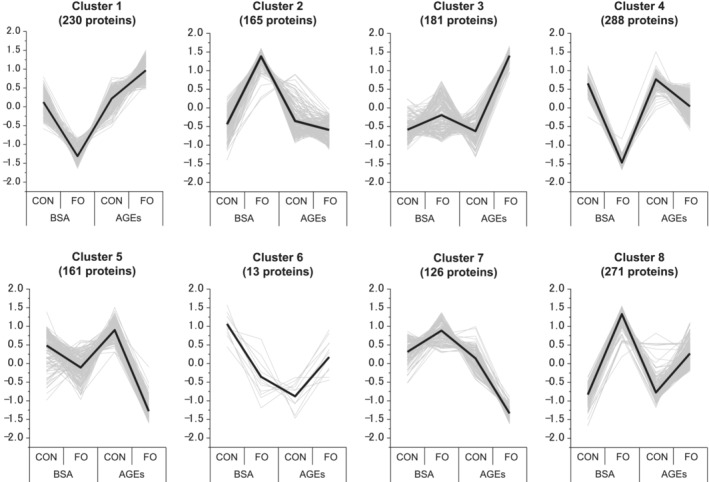
Clusters by K‐means clustering of significantly variable proteins. Bold lines indicate the means of each group. Grey lines indicate the level of dynamic patterns of proteins in each group. Vertical axis shows normalized expression changes (Z‐score).

Next, to extract significantly affected proteins, two‐way ANOVA was performed with functional overload (CON or FO) and AGE treatment (BSA or AGEs) as main factors. Consequently, 1435 proteins had significant interactions (*P* < 0.05). Subsequently, the 1435 proteins were clustered into eight using the K‐means method (Figure 5). Among these clusters, we selected clusters 2 and 8, which met the criteria for ‘clusters in which the Z‐score increased by more than 1 due to functional overload in BSA‐treated group (BSA‐CON vs. BSA‐FO) and the increase was attenuated by AGEs administration (BSA‐FO vs. AGEs‐FO)‘. The proteins of the two clusters may be involved in the mechanism underlying the muscle hypertrophy inhibition and muscle destruction by AGE treatment because the behaviour of the profile plots of the clusters corresponded to the result of the muscle weight (Figure 2) and CSA (Figure 4).

**Figure 6:**
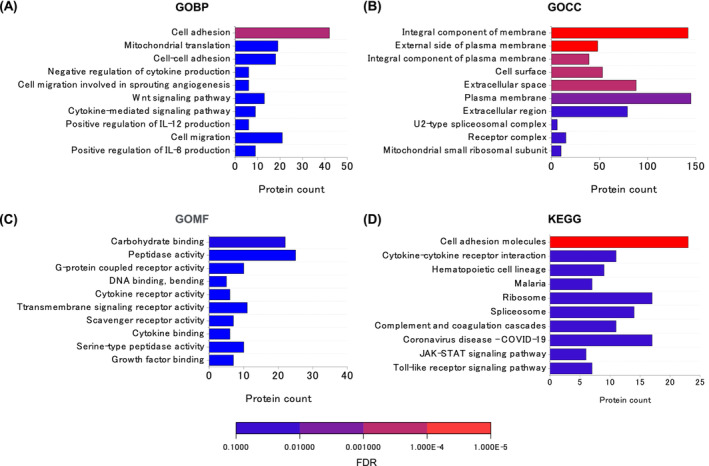
Functional Gene Ontology analysis of *(A)* GOBP, *(B)* GOCC, *(C)* GOMF, and *(D)* KEGG protein enrichment for proteins extracted using the K‐means clustering. The names of Gene Ontology ‘Biological Process’ (GOBP), Gene Ontology ‘Cellular Component’ (GOBP), Gene Ontology ‘Molecular Function’ (GOMF), and Kyoto Encyclopedia of Genes and Genomes (KEGG) pathway were used as functional annotations. Representative‐enriched GO terms are listed in ascending order of false discovery rate (FDR).

Then, for the 436 proteins in clusters 2 (165 proteins) and 8 (271 proteins), enrichment analysis on functional annotations was performed with the datasets of 4659 proteins as the background. The clusters were enriched in cell membrane integrity‐related terms, including ‘cell adhesion (GOBP)’, ‘integral component of membrane (GOCC)’, ‘integral component of plasma membrane (GOCC)’, and ‘cell adhesion molecules (KEGG)’ (Figure 6).

### Advanced glycation end products prevented overload‐induced increase of plasma membrane proteins in skeletal muscle

**Figure 7:**
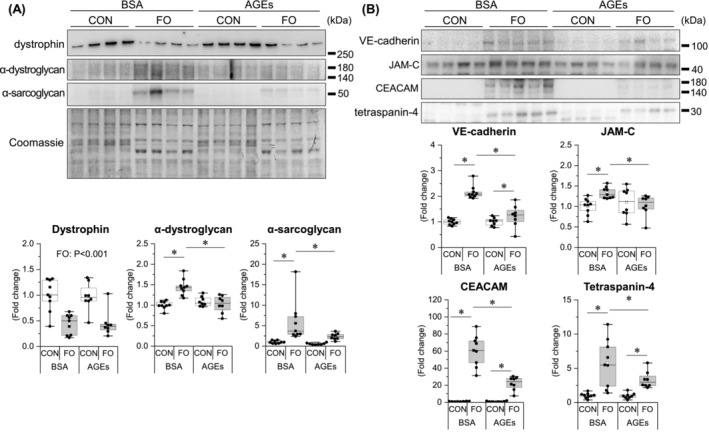
AGE administration prevents overload‐induced increase in skeletal muscle plasma membrane proteins. (A) Dystrophin–glycoprotein complex proteins. (B) Cell adhesion proteins. Data are shown as box plots. White square indicates mean values. *N* = 8–9 mice/group. Individual data points are indicated on the graph. Representative immunoblots are shown. Statistical significance is analysed using two‐way ANOVA with AGE treatment and overload surgery as main factors. *P < 0.05 with simple effects tests.

As proteomic analysis suggested the involvement of a group of proteins involved in cell membrane integrity, we examined changes in the expression of dystrophin–glycoprotein complex (DGC) proteins, which are responsible for cell membrane integrity, and cell adhesion‐related proteins identified by GO enrichment analysis (Tables S3–S5). DGC proteins, including α‐dystroglycan and α‐sarcoglycan (Figure 7A), and cell adhesion‐related proteins, including VE‐cadherin, JAM‐C (Figure 7B), CEACAM 1/2, and tetraspanin‐4 (Figure 7C), increased with functional overload but were attenuated by AGE treatment. Dystrophin was decreased by functional overloading independent of AGE treatment (Figure 7A).

### Advanced glycation end products impaired cell adhesion and membrane permeability in C2C12 muscle cells

**Figure 8:**
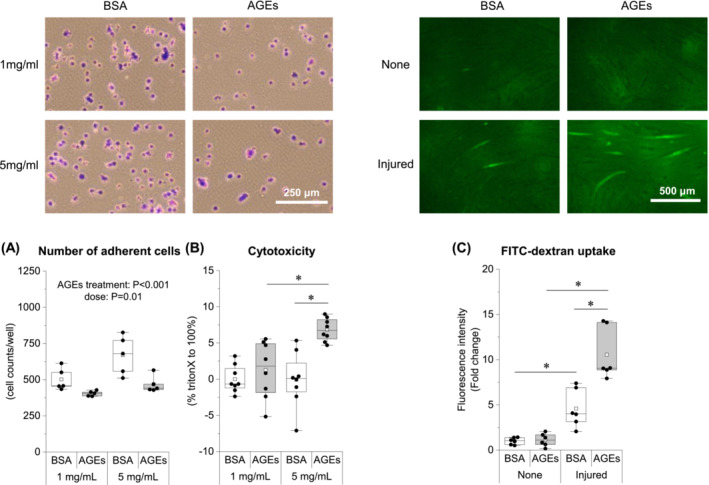
AGE treatment impairs cell adhesion and increases membrane permeability in C2C12 muscle cells. (A) Number of adherent cells observed 1 h after seeding with BSA or AGE‐stimulated cells. (B) Cytotoxicity by AGE stimulation. (C) FITC‐dextran uptake following glass beads injury to BSA‐ or AGE (1 mg/mL, 48 h)‐stimulated myotubes. Data are shown as box plots. White square indicates mean values. *N* = 5–8 mice/group. Individual data points are indicated on the graph. Representative immunofluorescence images are shown. Statistical significance is analysed using two‐way ANOVA with AGE treatment and overload surgery as main factors. *P < 0.05 with simple effects tests.

Next, we examined whether AGEs directly affect cell adhesion and impair cell membrane permeability using C2C12 muscle cells. The number of adherent cells was decreased by AGE treatment (Figure 8A) without cytotoxicity at 1 mg/mL concentration (Figure 8B). Membrane permeability was assessed by the uptake of FITC‐labelled dextran. FITC‐dextran is normally unable to cross the cell membrane and was used as a marker for changes in cell membrane permeability.^21^ Mechanical injury with glass beads increased the FITC‐dextran uptake, and the increase was more pronounced with pretreatment with AGEs (Figure 8C).

## Discussion

Glycative stress has been recognized as an inducer of skeletal muscle degenerative changes. AGE treatment inhibits myogenic capacity by suppressing myogenic regulatory factors in cultured muscle cells^22^, ^23^ and in growing mouse skeletal muscles.^6^ Diabetic muscle atrophy^11^ and disuse muscle atrophy^9^ were ameliorated by the administration of an AGE inhibitor and a RAGE antagonist, respectively. However, no studies have investigated the effect of glycative stress during muscle hypertrophic conditions. For the first time, we noted that high AGE levels in the blood attenuated overload‐induced muscle hypertrophy and an increase in protein synthesis (Figures 1, 2, and 3). In support of this result, our previous studies have shown that AGEs suppress protein synthesis signalling,^6^, ^7^ and AGEs may suppress muscle hypertrophy via the reduction of such pathways. Moreover, as it was accompanied by a decrease in myonuclear supply (Figure 3C), the reduced activity of muscle satellite cells may also be involved in muscle hypertrophy inhibition by AGEs.

An unexpected and interesting result of this study was the observed destruction of muscle fibres under overload in some AGE‐treated mice (Figure 3). Compensatory hypertrophy with synergist muscle ablation is a supraphysiological procedure that induces rapid muscle hypertrophy; however, this stimulation alone does not cause muscle fibre destruction,^24^ as in AGE‐unstimulated mice in our results (Figure 3). Therefore, it is likely that the muscle fibre destruction observed in this study was because of some changes in muscle cells induced by AGE treatment. PCA showed a clear difference between the AGE‐treated and non‐treated groups in the overloaded muscle (Figure 4). One possible cause of muscle fibre destruction is muscle fibre membrane weakening, as observed in muscle diseases, such as muscular dystrophy.^25^ Recent studies have shown that AGEs led to structural and functional changes in the meningeal membrane of the brain,^26^ increased placental vascular permeability,^27^ and affected cell adhesion in collagen fibrils.^28^ In support of these studies, our proteomic data also demonstrated the alteration of cell membrane integrity‐associated molecules by AGE treatment (Figure 6).

Sarcolemmal components, including DGC proteins, are responsible for membrane integrity, and the destabilization of these proteins leads to membrane fragility [S1]. An increase in regenerated muscle following muscle fibre destruction is observed in dystrophin‐deficient mdx mice^29^ and utrophin‐dystrophin–deficient mice.^30^ α‐sarcoglycan, which forms a heterotetramer with other sarcoglycans in the sarcolemma, is involved in maintaining the muscle contractile function, particularly in the fast‐twitch muscle used in this experiment,^31^ and its deficiency contributes to the progression of muscle degeneration.^32^ α‐dystroglycan serves as an anchor between the muscle cell membrane and the extracellular matrix, providing structural support and stability to the muscle fibres.^33^ Furthermore, DGC proteins play a role as mechanosensors during muscle hypertrophy stimulation.^34^ Our results showed that the expression changes of α‐sarcoglycan and α‐dystroglycan in response to hypertrophic stimuli were hindered by AGEs (Figure 7A), suggesting that it may have caused a decrease in muscle membrane integrity. To the authors' knowledge, this is the first study demonstrating that glycative stress affects DGC protein expression.

Additionally, cell adhesion‐related molecules, including VE‐cadherin, JAM‐C, CEACAM1, and tetraspanin‐4, showed similar changes to DGC (Figure 7B,C). The role of these molecules in the skeletal muscle is not fully elucidated; however, they are captured as molecules classified into clusters 2 or 8 (Figure 5) and are associated with membrane integrity (Tables S3–S5). VE‐cadherin is generally a component of adherens junctions between endothelial cells and plays a significant role in maintaining vascular integrity [S2]. JAM‐C is a cell surface receptor that is essential for muscle cell fusion [S3] and regulates vascular endothelial permeability by modulating VE‐cadherin–mediated adhesion [S4]. CEACAM1 is a cell adhesion molecule and facilitates endothelial cell migration via integrin binding [S5, S6]. The tetraspanin family is involved in several physiological processes in different organs, including cell adhesion, migration, immune response, fusion, and signal transduction [S7]; recently, the tetraspanin, CD82, has been shown to play a positive role in skeletal muscle regeneration by activating satellite cells [S8]. Based on these previous studies, a decreased expression of these molecules may have exacerbated the adhesion capacity and integrity of sarcolemmal membranes. It may also play a role in skeletal muscle capillary regression, considering the extensive evidence of vascular effects. The results of this study demonstrated that AGEs act directly on muscle cells to reduce cell adhesion and increase cell membrane permeability (Figure 8).

Loss of cell membrane integrity is a hallmark of skeletal muscle wasting in aging, diabetes, and cancer cachexia [S9, S10, S11]. Low DGC protein levels reduce lateral force transmission, increase sarcomere instability, and cause contraction‐induced muscle damage in aged rats.^35^ Furthermore, phospholipid composition reflects membrane fluidity and integrity.^36^ Evidence on the relationship between glycative stress and cell membrane function has indicated that the AGE–RAGE axis is involved in the impaired repair of muscle cell membranes associated with diabetes.^37^ A lifelong intervention study has shown that caloric restriction and voluntary exercise prevented aging‐related loss of muscle mass accompanied by DGC protein and membrane repair protein upregulation.^38^ Based on the abovementioned previous studies and our study, it is suggested that cell membrane fragility by AGEs may be a novel factor causing the onset of muscle dysfunctions and that improving cell membrane integrity by targeting glycative stress suppression may contribute to the prevention of skeletal muscle aging and diabetic muscle dysfunctions. Further studies are needed in animal models of aging, diabetes, and cancer cachexia to clarify this point. It is also necessary to investigate whether the AGE‐induced loss of cell membrane integrity affects muscle functions such as muscle tension and muscle endurance.

Accumulation of AGEs in the body progresses with age and metabolic diseases such as diabetes. There is a positive correlation between age and blood AGEs levels, and blood CML levels have been found to be several times higher in elderly people than in young people.^39^ Additionally, in diabetic patients, accumulation progresses additively, increasing up to about five times.^39^, ^40^ In the present study, blood and muscle AGEs levels were also several times higher in the AGEs‐treated group compared with the BSA‐treated group (Table S2). This suggests that physiological levels of AGEs in the body can have adverse effects on skeletal muscle. Furthermore, it is important to note that in this study, no obvious changes were observed when AGEs were stimulated alone, and the effects of AGEs were only observed when a strong overload stimulus was applied. This suggests that these changes may be more likely to occur in conditions of advanced inflammation, such as diabetes, considering that overload stimulation causes inflammation.

Previous research has shown that several types of AGEs are derived from glyceraldehyde, including glyceraldehyde‐derived pyridinium (GLAP), triosidines, MG‐H1, argpyrimidine, toxic AGEs (TAGE), and pyrrolopyridinium lysine dimer (PPG) [S12, S13, S14], but their bioactive effects are not fully understood. In this study, major AGEs were evaluated using ELISA, and it was confirmed that blood and muscle levels were elevated (Table S1). However, it is necessary to use mass spectrometry to accurately measure the levels and types of AGEs, which is a limitation in our research. Furthermore, because the glyceraldehyde concentration used to create AGEs was at the mM level, and even in diabetic patients it was at the μM level [S15], there is a possibility that unexpected AGE structures other than those mentioned above were generated in the human body. This point should be considered when interpreting this study.

In conclusion, the present study showed that AGE treatment prevented functional overload‐induced skeletal muscle hypertrophy by inhibiting protein synthesis in mice. Additionally, AGE treatment caused muscle fibre destruction accompanied by a decrease in DGC proteins and cell adhesion‐related proteins. AGE treatment to muscle cells also resulted in a loss of cell adhesion capacity and an increase in cell membrane permeability. These findings suggest that glycative stress may lead to a lack of membrane integrity and prevent muscle mass gain.

## Conflict of interest

The authors declare no conflict of interest.

## Supporting information


**Figure S1.** The data of grip strength and rotarod test. The grip strength test and rotarod test were performed the day before the end of the experiment. For grip strength test, mice were allowed to rest on the horizontal mesh with forelimb and hindlimb (four limb) and were then gently pulled back until their grip was broken. Grip strength was normalized to body weight and expressed as N/g. For rotarod test, after 30 s of adaptation at 5 rpm, the rotation speed was increased by 5 rpm every 30 s until the mouse fell, and the trial ended when the mouse falls. The rotation speed when it fell was recorded. Data are shown as box plots. White square indicates mean values. *n* = 8–9 mice/group. Individual data points are indicated on the graph. Statistical significance is analysed using t Mann–Whitney U‐test. * *P* < 0.05.
**Figure S2.** AGE administration did not affect protein synthesis signalling (mTOR and 4E‐BP1) in EDL muscle. Data are shown as box plots. White square indicates mean values. *n* = 6–9 mice/group. Individual data points are indicated on the graph. Representative immunoblots are shown. Statistical significance is analysed using two‐way ANOVA with AGE treatment and overload surgery as main factors. n.s., not significant.
**Figure S3.** AGE administration did not affect plasma membrane protein (α‐dystroglycan and α‐sarcoglycan) expressions in EDL muscle. Data are shown as box plots. White square indicates mean values. *n* = 4–8 mice/group. Individual data points are indicated on the graph. Representative immunoblots are shown. Statistical significance is analysed using two‐way ANOVA with AGE treatment and overload surgery as main factors. NS, not significant.
**Table S1.** AGEs concentration in administered substances.
**Table S2.** AGEs concentration in plasma and muscles.
**Table S3.** The list of proteins including functional annotation term ‘Cell adhesion (GOBP)’.
**Table S4.** The list of proteins including functional annotation term ‘Integral component of membrane (GOCC)’.
**Table S5.** The list of proteins including functional annotation term ‘Cell adhesion molecules (KEGG)’.
