# Supplementary material for: Glycative stress inhibits hypertrophy and impairs cell membrane integrity in overloaded mouse skeletal muscle
Source: J Cachexia Sarcopenia Muscle. 2024 Apr 4;15(3):883–96. doi: 10.1002/jcsm.13444 (PMC11154761; doi:10.1002/jcsm.13444)
Supplement: Supplementary file 1 — Figure S1. The data of grip strength and rotarod test. The grip strength test and rotarod test were performed the day before the end of the experiment. For grip strength test, mice were allowed to rest on the horizontal mesh with forelimb and hindlimb (four limb) and were then gently pulled back until their grip was broken. Grip strength was normalized to body weight and expressed as N/g. For rotarod test, after 30 s of adaptation at 5 rpm, the rotation speed was increased by 5 rpm every 30 s until the mouse fell, and the trial ended when the mouse falls. The rotation speed when it fell was recorded. Data are shown as box plots. White square indicates mean values. n = 8–9 mice/group. Individual data points are indicated on the graph. Statistical significance is analysed using t Mann–Whitney U‐test. * P < 0.05. Figure S2. AGE administration did not affect protein synthesis signalling (mTOR and 4E‐BP1) in EDL muscle. Data are shown as box plots. White square indicates mean values. n = 6–9 mice/group. Individual data points are indicated on the graph. Representative immunoblots are shown. Statistical significance is analysed using two‐way ANOVA with AGE treatment and overload surgery as main factors. n.s., not significant. Figure S3. AGE administration did not affect plasma membrane protein (α‐dystroglycan and α‐sarcoglycan) expressions in EDL muscle. Data are shown as box plots. White square indicates mean values. n = 4–8 mice/group. Individual data points are indicated on the graph. Representative immunoblots are shown. Statistical significance is analysed using two‐way ANOVA with AGE treatment and overload surgery as main factors. NS, not significant. Table S1. AGEs concentration in administered substances. Table S2. AGEs concentration in plasma and muscles. Table S3. The list of proteins including functional annotation term ‘Cell adhesion (GOBP)’. Table S4. The list of proteins including functional annotation term ‘Integral component of membrane (GOCC)’. Table S5. [file JCSM-15-883-s001.docx]

**Supplementary Figure 1**


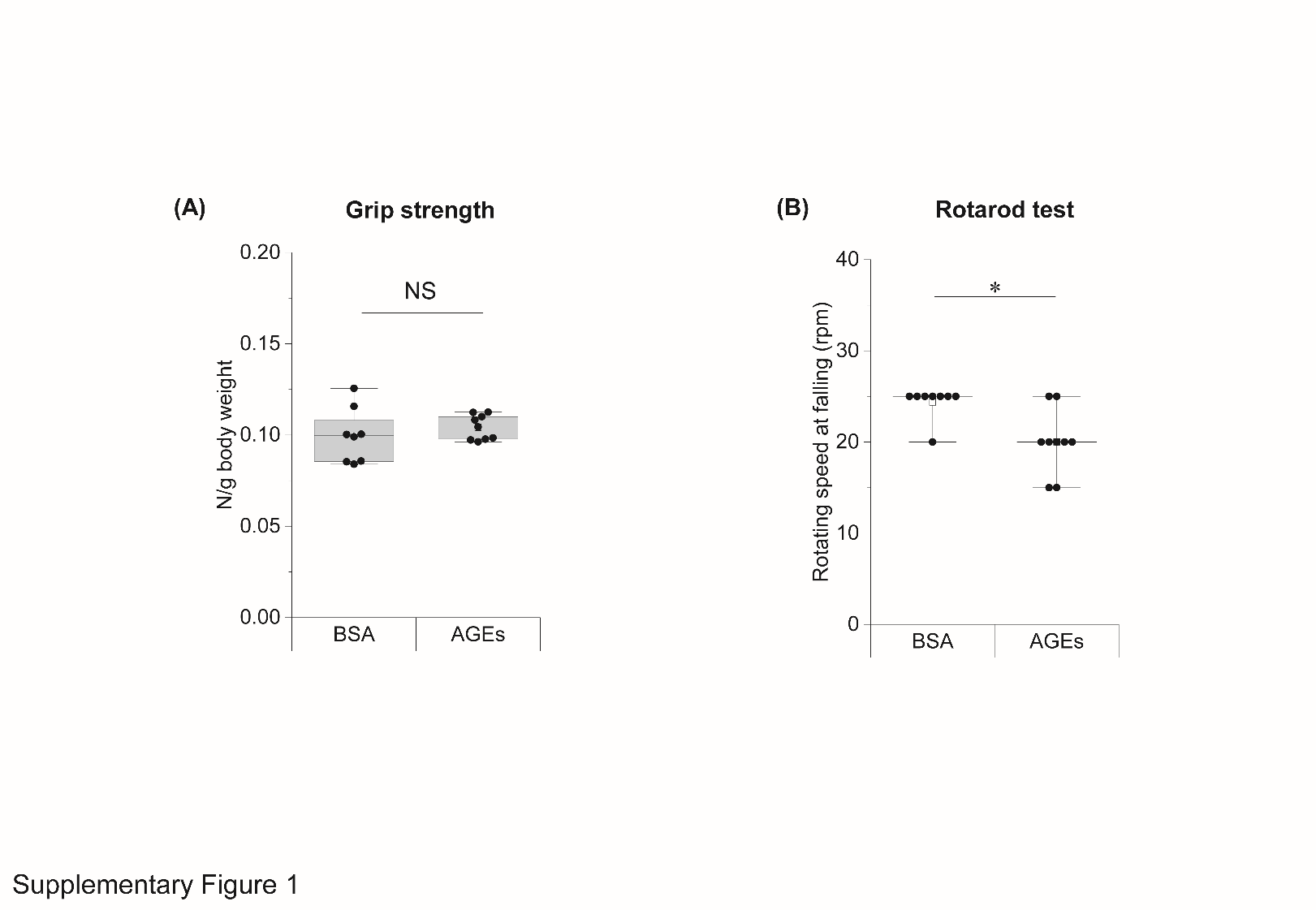


The data of grip strength and rotarod test. The grip strength test and rotarod test were performed the day before the end of the experiment. For grip strength test, mice were allowed to rest on the horizontal mesh with forelimb and hindlimb (four limb) and were then gently pulled back until their grip was broken. Grip strength was normalized to body weight and expressed as N/g. For rotarod test, after 30 s of adaptation at 5 rpm, the rotation speed was increased by 5 rpm every 30 s until the mouse fell, and the trial ended when the mouse falls. The rotation speed when it fell was recorded. Data are shown as box plots. White square indicates mean values. n = 8–9 mice/group. Individual data points are indicated on the graph. Statistical significance is analyzed using t Mann-Whitney U-test. * P < 0.05.

**Supplementary Figure 2**


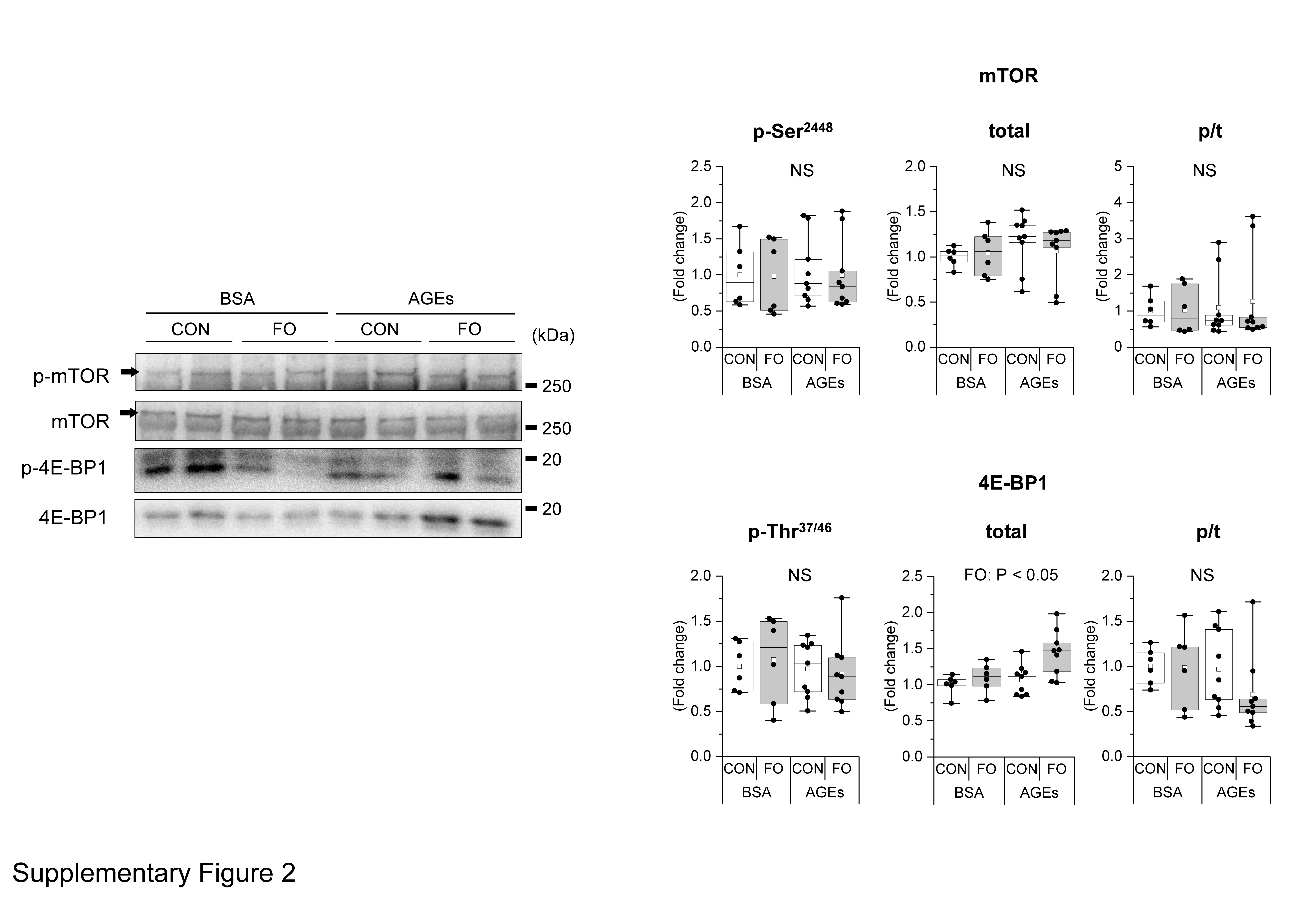


AGE administration did not affect protein synthesis signaling (mTOR and 4E-BP1) in EDL muscle. Data are shown as box plots. White square indicates mean values. n = 6–9 mice/group. Individual data points are indicated on the graph. Representative immunoblots are shown. Statistical significance is analyzed using two-way ANOVA with AGE treatment and overload surgery as main factors. n.s., not significant.

**Supplementary Figure 3**


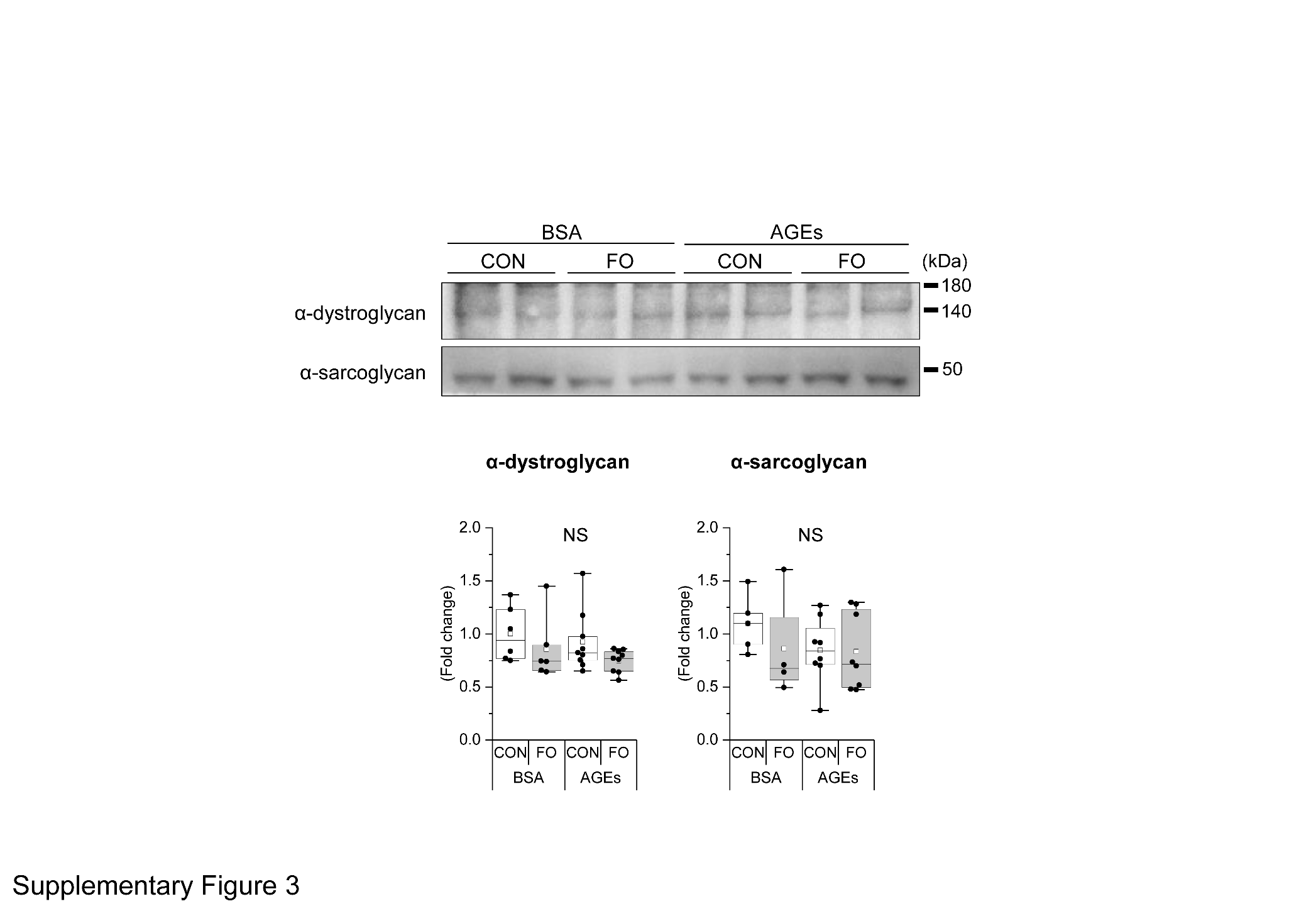


AGE administration did not affect plasma membrane protein (α-dystroglycan and α-sarcoglycan) expressions in EDL muscle. Data are shown as box plots. White square indicates mean values. n = 4–8 mice/group. Individual data points are indicated on the graph. Representative immunoblots are shown. Statistical significance is analyzed using two-way ANOVA with AGE treatment and overload surgery as main factors. NS, not significant.

**Supplementary Table 1. AGEs concentration in administered substances**

|  | BSA | AGEs |
| --- | --- | --- |
| Fluorescence AGEs (AU) | 2.51 ± 1.5 | 91.0 ± 3.5 |
| CML (μg/mL) | ND | 242.7± 3.1 |
| CEL (μg/mL) | ND | 59.1 ± 0.9 |
| MG-H1 (μg/mL) | ND | 65.5 ± 0.02 |

Values are mean ± SD, n = 3/groups. AU, arbitrary unit; ND, not detected; CML, Nε-(Carboxymethyl)lysine; CEL, Nε-(Carboxyethyl)lysine; MG-H1, methylglyoxal-derived hydroimidazolone-1.

**Supplementary Table 2. AGEs concentration in plasma and muscles**

|  | Day 0 | | Day 7 | | main effect of AGEs  (ANOVA) |
| --- | --- | --- | --- | --- | --- |
|  | BSA | AGEs | BSA | AGEs |  |
| Plasma | | | | | |
| Fluorescence AGEs  (AU) | 4.7 ± 0.3 | 8.3 ± 1.1 | 4.0 ± 0.6 | 8.1 ± 0.2 | P < 0.001 |
| CML  (μg/mL) | 0.81 ± 0.81 | 1.83 ± 0.85 | 1.61 ± 0.59 | 1.79 ± 0.60 | P = 0.044 |
| CEL  (μg/mL) | 0.26 ± 0.24 | 0.69 ± 0.15 | 0.36 ± 0.12 | 0.51 ± 0.21 | P < 0.001 |
| MG-H1  (μg/mL) | 0.15 ± 0.06 | 0.61 ± 0.10 | 0.20 ± 0.02 | 0.51 ± 0.14 | P < 0.001 |
| PLA muscle | | | | | |
| Fluorescence AGEs  (AU) | 1.7 ± 0.8 | 1.8 ± 0.5 | 1.8 ± 0.8 | 2.0 ± 0.7 | P < 0.001 |
| CML  (μg/mg protein) | 0.67 ± 0.24 | 1.05 ± 0.41 | 0.78 ± 0.23 | 0.96 ± 0.30 | P = 0.012 |
| CEL  (μg/mg protein) | 0.75 ± 0.38 | 0.68 ± 0.10 | 0.55 ± 0.09 | 0.61 ± 0.10 | NS |
| MG-H1  (μg/mg protein) | 0.09 ± 0.01 | 0.18 ± 0.02 | 0.07 ± 0.01 | 0.30 ± 0.11 | P < 0.001 |

Values are mean ± SD, n = 6-9/groups. AU, arbitrary unit; NS, not significant; CML, Nε-(Carboxymethyl)lysine; CEL, Nε-(Carboxyethyl)lysine; MG-H1, methylglyoxal-derived hydroimidazolone-1.

**Supplementary Table 3. The list of proteins including functional annotation term “Cell adhesion (GOBP)”.**

| **ID** | **Protein Name** |
| --- | --- |
| Q64314 | CD34 antigen (Cd34) |
| P15379 | CD44 antigen (Cd44) |
| O89103 | CD93 antigen (Cd93) |
| P31809 | CEA cell adhesion molecule 1 (Ceacam1) |
| O88792 | F11 receptor (F11r) |
| Q8BKG3 | PTK7 protein tyrosine kinase 7 (Ptk7) |
| Q61824 | a disintegrin and metallopeptidase domain 12 (meltrin alpha) (Adam12) |
| O88839 | a disintegrin and metallopeptidase domain 15 (metargidin) (Adam15) |
| Q61490 | activated leukocyte cell adhesion molecule (Alcam) |
| Q61282 | aggrecan (Acan) |
| Q64726 | alpha-2-glycoprotein 1, zinc (Azgp1) |
| P12023 | amyloid beta (A4) precursor protein (App) |
| P15116 | cadherin 2 (Cdh2) |
| P55284 | cadherin 5 (Cdh5) |
| Q8R1U2 | cell growth regulator with EF hand domain 1 (Cgref1) |
| Q64299 | cellular communication network factor 3 (Ccn3) |
| O54775 | cellular communication network factor 4 (Ccn4) |
| Q02788 | collagen, type VI, alpha 2 (Col6a2) |
| P28843 | dipeptidylpeptidase 4 (Dpp4) |
| P21995 | embigin (Emb) |
| Q925F2 | endothelial cell-specific adhesion molecule (Esam) |
| P52800 | ephrin B2 (Efnb2) |
| Q2VIS4 | filaggrin family member 2 (Flg2) |
| O35930 | glycoprotein 1b, alpha polypeptide (Gp1ba) |
| O88186 | glycoprotein 9 (platelet) (Gp9) |
| Q3V3R4 | integrin alpha 1 (Itga1) |
| P61622 | integrin alpha 11 (Itga11) |
| P05555 | integrin alpha M (Itgam) |
| P09055 | integrin beta 1 (fibronectin receptor beta) (Itgb1) |
| P35330 | intercellular adhesion molecule 2 (Icam2) |
| Q9D8B7 | junction adhesion molecule 3 (Jam3) |
| A6H6E2 | multimerin 2 (Mmrn2) |
| Q9JKF6 | nectin cell adhesion molecule 1 (Nectin1) |
| Q08481 | platelet/endothelial cell adhesion molecule 1 (Pecam1) |
| P28828 | protein tyrosine phosphatase, receptor type, M (Ptprm) |
| Q7TQ33 | repulsive guidance molecule family member B (Rgmb) |
| P18337 | selectin, lymphocyte (Sell) |
| Q01102 | selectin, platelet (Selp) |
| Q920G3 | sialic acid binding Ig-like lectin F (Siglecf) |
| Q8K007 | sulfatase 1 (Sulf1) |
| A2AVA0 | sushi, von Willebrand factor type A, EGF and pentraxin domain containing 1 (Svep1) |
| P29533 | vascular cell adhesion molecule 1 (Vcam1) |

**Supplementary Table 4. The list of proteins including functional annotation term “Integral component of membrane (GOCC)”.**

| **ID** | **Protein Name** |
| --- | --- |
| P49300 | C-type lectin domain family 10, member A (Clec10a) |
| Q8VCP9 | C-type lectin domain family 14, member a (Clec14a) |
| Q9QZ15 | C-type lectin domain family 4, member a2 (Clec4a2) |
| Q6QLQ4 | C-type lectin domain family 7, member a (Clec7a) |
| Q2VLH6 | CD163 antigen (Cd163) |
| Q91ZW8 | CD209d antigen (Cd209d) |
| Q91V98 | CD248 antigen, endosialin (Cd248) |
| Q8VE98 | CD276 antigen (Cd276) |
| Q64314 | CD34 antigen (Cd34) |
| P15379 | CD44 antigen (Cd44) |
| P42082 | CD86 antigen (Cd86) |
| O89103 | CD93 antigen (Cd93) |
| P31809 | CEA cell adhesion molecule 1 (Ceacam1) |
| O88792 | F11 receptor (F11r) |
| Q61559 | Fc fragment of IgG receptor and transporter (Fcgrt) |
| Q6Y7W8 | GRB10 interacting GYF protein 2 (Gigyf2) |
| P42703 | LIF receptor alpha (Lifr) |
| Q69ZN6 | N-acetylglucosamine-1-phosphate transferase, alpha and beta subunits (Gnptab) |
| Q8BKG3 | PTK7 protein tyrosine kinase 7 (Ptk7) |
| Q8K201 | RIKEN cDNA 9530068E07 gene (9530068E07Rik) |
| Q11204 | ST3 beta-galactoside alpha-2,3-sialyltransferase 2 (St3gal2) |
| Q91Y74 | ST3 beta-galactoside alpha-2,3-sialyltransferase 4 (St3gal4) |
| Q8CI59 | STEAP family member 3 (Steap3) |
| Q3TDQ1 | STT3, subunit of the oligosaccharyltransferase complex, homolog B (S. cerevisiae) (Stt3b) |
| Q99MB3 | TM2 domain containing 1 (Tm2d1) |
| Q8R0I4 | TM2 domain containing 2 (Tm2d2) |
| Q920V1 | UDP-GalNAc:betaGlcNAc beta 1,3-galactosaminyltransferase, polypeptide 1 (B3galnt1) |
| D3YX43 | V-set and immunoglobulin domain containing 10 (Vsig10) |
| Q61824 | a disintegrin and metallopeptidase domain 12 (meltrin alpha) (Adam12) |
| O88839 | a disintegrin and metallopeptidase domain 15 (metargidin) (Adam15) |
| Q61490 | activated leukocyte cell adhesion molecule (Alcam) |
| G5E8Q8 | adhesion G protein-coupled receptor F5 (Adgrf5) |
| Q8CJ12 | adhesion G protein-coupled receptor G2 (Adgrg2) |
| Q8JZZ7 | adhesion G protein-coupled receptor L2 (Adgrl2) |
| P12023 | amyloid beta (A4) precursor protein (App) |
| P35374 | angiotensin II receptor, type 2 (Agtr2) |
| Q9WU60 | attractin (Atrn) |
| Q8R2Q8 | bone marrow stromal cell antigen 2 (Bst2) |
| P15116 | cadherin 2 (Cdh2) |
| P55284 | cadherin 5 (Cdh5) |
| Q9QUP4 | carbohydrate (N-acetylglucosamine 6-O) sulfotransferase 5 (Chst5) |
| Q99LL3 | carbohydrate sulfotransferase 12 (Chst12) |
| O89001 | carboxypeptidase D (Cpd) |
| P51675 | chemokine (C-C motif) receptor 1 (Ccr1) |
| Q9CQX5 | claudin domain containing 1 (Cldnd1) |
| Q9D162 | coiled-coil domain containing 167 (Ccdc167) |
| Q8K4Q8 | collectin sub-family member 12 (Colec12) |
| P09581 | colony stimulating factor 1 receptor (Csf1r) |
| Q64735 | complement component (3b/4b) receptor 1-like (Cr1l) |
| P00158 | cytochrome b, mitochondrial (mt-Cytb) |
| Q91W29 | cytochrome c oxidase subunit 4I2 (Cox4i2) |
| P28843 | dipeptidylpeptidase 4 (Dpp4) |
| Q3UMY5 | echinoderm microtubule associated protein like 4 (Eml4) |
| Q8VD58 | ecotropic viral integration site 2 (Evi2) |
| Q8VD58 | ecotropic viral integration site 2b (Evi2b) |
| P55772 | ectonucleoside triphosphate diphosphohydrolase 1 (Entpd1) |
| Q6DYE8 | ectonucleotide pyrophosphatase/phosphodiesterase 3 (Enpp3) |
| P21995 | embigin (Emb) |
| Q3TZW0 | endothelial cell surface expressed chemotaxis and apoptosis regulator (Ecscr) |
| Q925F2 | endothelial cell-specific adhesion molecule (Esam) |
| Q4PZA2 | endothelin converting enzyme 1 (Ece1) |
| P52800 | ephrin B2 (Efnb2) |
| Q8K135 | expressed sequence AU040320 (AU040320) |
| Q8C0Z1 | family with sequence similarity 234, member A (Fam234a) |
| Q9JHJ3 | glycosylated lysosomal membrane protein (Glmp) |
| P70387 | homeostatic iron regulator (Hfe) |
| Q6ZQA6 | immunoglobulin superfamily, member 3 (Igsf3) |
| Q3V3R4 | integrin alpha 1 (Itga1) |
| Q99KW9 | integrin alpha FG-GAP repeat containing 1 (Itfg1) |
| P05555 | integrin alpha M (Itgam) |
| O35664 | interferon (alpha and beta) receptor 2 (Ifnar2) |
| Q61730 | interleukin 1 receptor accessory protein (Il1rap) |
| Q61190 | interleukin 10 receptor, beta (Il10rb) |
| Q00560 | interleukin 6 signal transducer (Il6st) |
| P35918 | kinase insert domain protein receptor (Kdr) |
| Q8C129 | leucyl/cystinyl aminopeptidase (Lnpep) |
| O08843 | leukocyte specific transcript 1 (Lst1) |
| Q8BG84 | leukocyte-associated Ig-like receptor 1 (Lair1) |
| P35951 | low density lipoprotein receptor (Ldlr) |
| P30204 | macrophage scavenger receptor 1 (Msr1) |
| Q8CBH5 | major facilitator superfamily domain containing 6 (Mfsd6) |
| O09159 | mannosidase 2, alpha B1 (Man2b1) |
| Q8R4G6 | mannoside acetylglucosaminyltransferase 5 (Mgat5) |
| Q9D8U6 | mast cell expressed membrane protein 1 (Mcemp1) |
| Q61391 | membrane metallo endopeptidase (Mme) |
| O35682 | myeloid-associated differentiation marker (Myadm) |
| Q9JKF6 | nectin cell adhesion molecule 1 (Nectin1) |
| Q8CJ26 | neurotrophin receptor associated death domain (Nradd) |
| P57716 | nicastrin (Ncstn) |
| O70458 | oncostatin M receptor (Osmr) |
| Q99LV7 | phosphatidylinositol glycan anchor biosynthesis, class X (Pigx) |
| Q62028 | phospholipase A2 receptor 1 (Pla2r1) |
| Q8R143 | pituitary tumor-transforming 1 interacting protein (Pttg1ip) |
| Q8VIK5 | platelet endothelial aggregation receptor 1 (Pear1) |
| Q08481 | platelet/endothelial cell adhesion molecule 1 (Pecam1) |
| Q9DC11 | plexin domain containing 2 (Plxdc2) |
| O08852 | polycystin 1, transient receptor potential channel interacting (Pkd1) |
| Q8C102 | polypeptide N-acetylgalactosaminyltransferase 5 (Galnt5) |
| Q9WV91 | prostaglandin F2 receptor negative regulator (Ptgfrn) |
| P43252 | prostaglandin I receptor (IP) (Ptgir) |
| Q64695 | protein C receptor, endothelial (Procr) |
| B2RU80 | protein tyrosine phosphatase, receptor type, B (Ptprb) |
| Q05909 | protein tyrosine phosphatase, receptor type, G (Ptprg) |
| P28828 | protein tyrosine phosphatase, receptor type, M (Ptprm) |
| Q3TUA9 | protein-O-mannose kinase (Pomk) |
| Q9D771 | proton activated chloride channel 1 (Pacc1) |
| Q9CZQ6 | retinoic acid early transcript 1E (Raet1e) |
| O54965 | ring finger protein 13 (Rnf13) |
| Q5DTZ6 | ring finger protein 150 (Rnf150) |
| Q8C850 | scavenger receptor class A, member 3 (Scara3) |
| Q8K299 | scavenger receptor class A, member 5 (Scara5) |
| Q61009 | scavenger receptor class B, member 1 (Scarb1) |
| O35114 | scavenger receptor class B, member 2 (Scarb2) |
| P18337 | selectin, lymphocyte (Sell) |
| Q01102 | selectin, platelet (Selp) |
| Q920G3 | sialic acid binding Ig-like lectin F (Siglecf) |
| Q9JLZ8 | single immunoglobulin and toll-interleukin 1 receptor (TIR) domain (Sigirr) |
| Q3UTD9 | small integral membrane protein 15 (Smim15) |
| P51912 | solute carrier family 1 (neutral amino acid transporter), member 5 (Slc1a5) |
| Q8K4D3 | solute carrier family 36 (proton/amino acid symporter), member 1 (Slc36a1) |
| Q6PHU5 | sortilin 1 (Sort1) |
| O09117 | synaptophysin-like protein (Sypl) |
| P43407 | syndecan 2 (Sdc2) |
| O35988 | syndecan 4 (Sdc4) |
| Q9EP64 | tenomodulin (Tnmd) |
| Q9DCK3 | tetraspanin 4 (Tspan4) |
| O70401 | tetraspanin 6 (Tspan6) |
| Q8C0L0 | thioredoxin-related transmembrane protein 4 (Tmx4) |
| Q99MB1 | toll-like receptor 3 (Tlr3) |
| Q9QUK6 | toll-like receptor 4 (Tlr4) |
| P58682 | toll-like receptor 8 (Tlr8) |
| Q62314 | trans-golgi network protein 2 (Tgoln2) |
| Q62313 | trans-golgi network protein (Tgoln1) |
| Q8VC04 | transmembrane protein 106A (Tmem106a) |
| Q80X71 | transmembrane protein 106B (Tmem106b) |
| Q8C4Q9 | transmembrane protein 154 (Tmem154) |
| Q8CIB6 | transmembrane protein 230 (Tmem230) |
| Q9DAM7 | transmembrane protein 263 (Tmem263) |
| Q8VEK0 | transmembrane protein 30A (Tmem30a) |
| Q8BXN9 | transmembrane protein 87A (Tmem87a) |
| P25119 | tumor necrosis factor receptor superfamily, member 1b (Tnfrsf1b) |
| P29533 | vascular cell adhesion molecule 1 (Vcam1) |

**Supplementary Table 5. The list of proteins including functional annotation term “Cell adhesion molecules (KEGG)”.**

| **ID** | **Protein Name** |
| --- | --- |
| Q8VE98 | CD276 antigen (Cd276) |
| Q64314 | CD34 antigen (Cd34) |
| P27512 | CD40 antigen (Cd40) |
| P42082 | CD86 antigen (Cd86) |
| O88792 | F11 receptor (F11r) |
| Q61490 | activated leukocyte cell adhesion molecule (Alcam) |
| P15116 | cadherin 2 (Cdh2) |
| P55284 | cadherin 5 (Cdh5) |
| Q925F2 | endothelial cell-specific adhesion molecule (Esam) |
| P01898 | histocompatibility 2, Q region locus 10 (H2-Q10) |
| P05555 | integrin alpha M (Itgam) |
| P09055 | integrin beta 1 (fibronectin receptor beta) (Itgb1) |
| P35330 | intercellular adhesion molecule 2 (Icam2) |
| Q9D8B7 | junction adhesion molecule 3 (Jam3) |
| Q3TEW6 | myelin protein zero-like 1 (Mpzl1) |
| Q9JKF6 | nectin cell adhesion molecule 1 (Nectin1) |
| Q08481 | platelet/endothelial cell adhesion molecule 1 (Pecam1) |
| P28828 | protein tyrosine phosphatase, receptor type, M (Ptprm) |
| P18337 | selectin, lymphocyte (Sell) |
| Q01102 | selectin, platelet (Selp) |
| P43407 | syndecan 2 (Sdc2) |
| O35988 | syndecan 4 (Sdc4) |
| P29533 | vascular cell adhesion molecule 1 (Vcam1) |

**Supplementary references**

S1. Gumerson JD, Michele DE. The dystrophin-glycoprotein complex in the prevention of muscle damage. J Biomed Biotechnol. 2011;2011:210797.

S2. Giannotta M, Trani M, Dejana E. VE-cadherin and endothelial adherens junctions: active guardians of vascular integrity. Dev Cell. 2013;26:441-54.

S3. Powell GT, Wright GJ. Jamb and jamc are essential for vertebrate myocyte fusion. PLoS Biol. 2011;9:e1001216.

S4. Orlova VV, Economopoulou M, Lupu F, Santoso S, Chavakis T. Junctional adhesion molecule-C regulates vascular endothelial permeability by modulating VE-cadherin-mediated cell-cell contacts. J Exp Med. 2006;203:2703-14.

S5. Muller MM, Singer BB, Klaile E, Obrink B, Lucka L. Transmembrane CEACAM1 affects integrin-dependent signaling and regulates extracellular matrix protein-specific morphology and migration of endothelial cells. Blood. 2005;105:3925-34.

S6. Brummer J, Ebrahimnejad A, Flayeh R, Schumacher U, Loning T, Bamberger AM, et al. cis Interaction of the cell adhesion molecule CEACAM1 with integrin beta(3). Am J Pathol. 2001;159:537-46.

S7. Hemler ME. Tetraspanin functions and associated microdomains. Nat Rev Mol Cell Biol. 2005;6:801-11.

S8. Hall A, Fontelonga T, Wright A, Bugda Gwilt K, Widrick J, Pasut A, et al. Tetraspanin CD82 is necessary for muscle stem cell activation and supports dystrophic muscle function. Skelet Muscle. 2020;10:34.

S9. Demontis F, Piccirillo R, Goldberg AL, Perrimon N. Mechanisms of skeletal muscle aging: insights from Drosophila and mammalian models. Dis Model Mech. 2013;6:1339-52.

S10. Acharyya S, Butchbach ME, Sahenk Z, Wang H, Saji M, Carathers M, et al. Dystrophin glycoprotein complex dysfunction: a regulatory link between muscular dystrophy and cancer cachexia. Cancer Cell. 2005;8:421-32.

S11. Aleksandrowicz R, Straczkowski M. Link between insulin resistance and skeletal muscle extracellular matrix remodeling. Endocr Connect. 2023;12:e230023.

S12. Shigeta T, Sasamoto K, Yamamoto T. Glyceraldehyde-derived advanced glycation end-products having pyrrolopyridinium-based crosslinks. Biochem Biophys Rep. 2021;26:100963.

S13. Takata T, Masauji T, Motoo Y. Analysis of Crude, Diverse, and Multiple Advanced Glycation End-Product Patterns May Be Important and Beneficial. Metabolites. 2024;14:3.

S14. Takeuchi M, Sakasai-Sakai A, Takata T, Takino JI, Koriyama Y. Effects of Toxic AGEs (TAGE) on Human Health. Cells. 2022;11:

S15. Martin-Morales A, Arakawa T, Sato M, Matsumura Y, Mano-Usui F, Ikeda K, et al. Development of a Method for Quantitation of Glyceraldehyde in Various Body Compartments of Rodents and Humans. J Agric Food Chem. 2021;69:13246-54.
